# Therapeutic Targeting of the Janus Kinase/Signal Transducer and Activator of Transcription Pathway in Cutaneous T-Cell Lymphoma

**DOI:** 10.3390/cancers17040568

**Published:** 2025-02-07

**Authors:** Alisha Kashyap, Julia Dai, Xiao Ni

**Affiliations:** 1 John P. and Kathrine G. McGovern Medical School at the University of Texas Health Science Center at Houston, Houston, TX 77030, USA; 2 Department of Dermatology, The University of Texas MD Anderson Cancer Center, Houston, TX 77030, USA

**Keywords:** cutaneous T-cell lymphoma, mycosis fungoides, Sézary syndrome, pathogenesis, JAK/STAT pathway, therapeutic strategies, JAK/STAT inhibitors, clinical response, adverse effects

## Abstract

**Background/Objectives**: Cutaneous T-cell lymphoma (CTCL) is a rare group of non-Hodgkin lymphomas characterized by the clonal expansion of malignant T cells. While current treatments can alleviate symptoms and significant progress has been made in treating leukemic CTCL, a definitive cure remains elusive. Dysregulation of the Janus kinase/signal transducer and activator of transcription (JAK/STAT) signaling pathway is a key driver of CTCL pathogenesis. As a result, therapeutic strategies targeting JAK/STAT signaling have gained momentum, with the increasing use of JAK inhibitors and other agents that effectively suppress this pathway. These immune-modulating therapies have broad effects on physiological processes, inflammation, and the pathological changes associated with both inflammatory diseases and cancers. Several JAK inhibitors, originally FDA-approved for inflammatory conditions, are now being investigated for cancer treatment. **Methods**: In this paper, a brief review of the literature on JAK/STAT pathway dysregulation in CTCL is provided, highlighting both clinical and preclinical studies involving JAK inhibitors and other agents that target this pathway. **Results**: Specifically, we focus on six JAK inhibitors currently under clinical investigation—golidocitinib, ruxolitinib, cerdulatinib, tofacitinib, upadacitinib, and abrocitinib. Additionally, we discuss preclinical studies that explore the mechanisms underlying JAK/STAT pathway inhibition in CTCL. Furthermore, we review reported cases in which CTCL relapsed or emerged following JAK inhibitor treatment. **Conclusions**: Collectively, these findings support the potential clinical utility of targeting the JAK/STAT pathway in CTCL. However, further research is needed to evaluate safety risks, minimize adverse effects, and optimize these therapeutic strategies.

## Simple Summary

Cutaneous T-cell lymphoma (CTCL) is a rare cancer of immune cells in the skin. Current treatments help manage the symptoms, but there is not yet a cure. A key issue in CTCL is the abnormal activity of the JAK/STAT pathway, which controls the signals that help cells grow and survive. This pathway is often overactive in CTCL, contributing to the disease’s progression. Recently, a new group of drugs called JAK inhibitors, which block this pathway, have shown promise in treating CTCL. Some of these drugs, like ruxolitinib and cerdulatinib, are already used for inflammatory diseases, and early studies have suggested they might also help CTCL patients with manageable side effects. However, there are concerns that JAK inhibitors could potentially worsen or even trigger CTCL in some cases. Scientists are conducting laboratory studies to better understand how these drugs work and to identify which patients might benefit the most. While more research is needed, targeting this pathway could offer new hope for CTCL patients by providing a more effective treatment option.

## 1. Introduction

Cutaneous T-cell lymphoma (CTCL) is a rare subtype of non-Hodgkin lymphoma with an annual incidence of 0.5 per 100,000 [1]. CTCL encompasses a diverse group of cutaneous lymphoproliferative disorders (LPDs) characterized by clonal malignant T-cell proliferation in the skin. According to the WHO-EORTC classification, CTCL includes mycosis fungoides (MF), MF variants, Sézary syndrome (SS), adult T-cell leukemia/lymphoma (ATLL), primary cutaneous CD30^+^ LPDs, subcutaneous panniculitis-like T-cell lymphoma (SPTCL), extranodal NK/T-cell lymphoma-nasal type (ENKTCL-NT), chronic active EBV infection, primary cutaneous peripheral T-cell lymphoma (PTCL)-rare subtypes, and primary cutaneous PTCL-not otherwise specified (PTCL-NOS). MF and SS are the classic types of CTCL and account for 60% of all cases [2]. The management strategies for CTCL depend on factors such as the extent of skin disease, severity of extracutaneous involvement, and symptomatology. The treatment options range widely, spanning skin-directed therapies, including topical steroids, topical nitrogen mustard, phototherapy, and radiotherapy, to systemic approaches, such as immune modulators, retinoids, antimetabolites, targeted antibody therapy, and allogeneic hematopoietic stem cell transplantation [3]. While current treatments can alleviate symptoms and significant progress has been made in treating leukemic CTCL, a definitive cure for CTCL remains elusive.

**Figure 1:**
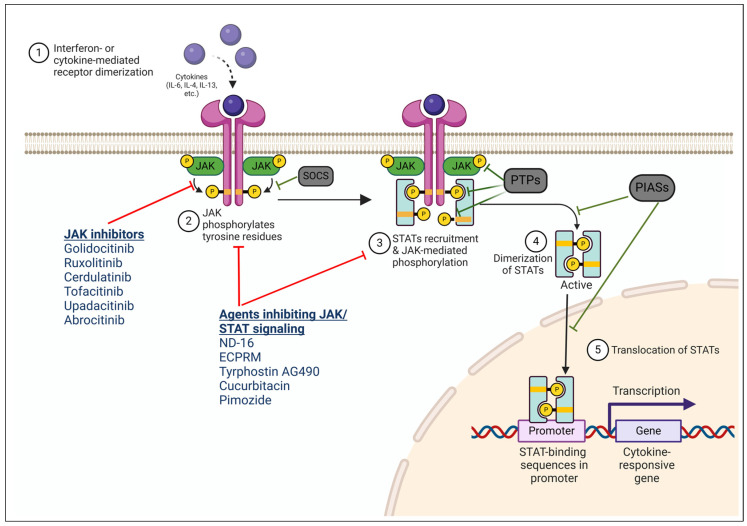
The diagram of the cytokine-mediated JAK/STAT signaling pathway and potential targets of inhibition of the pathway. ① Upon binding of cytokines to the cytokine receptors, the signal transduction process is initiated. Cytokine-mediated receptor dimerization leads to ② the recruitment and activation of JAKs by phosphorylating tyrosine residues; ③ Activated JAKs recruit and phosphorylate STATs, ④ that then dimerize and ⑤ translocate to the nucleus. In the nucleus, STATs bind to STAT-binding promoter in the DNA, leading to the transcription of cytokine responsive genes. There are a few negative regulators along the JAK/STAT signaling pathway: suppressors of cytokine signaling proteins (SOCS) inhibit JAKs by acting as pseudo-JAK substrates, while protein tyrosine phosphatases (PTPs) down-regulate the pathway by dephosphorylating JAK/STAT components; protein inhibitors of activated STATs (PIASs) can interact with activated STATs and inhibit their DNA binding and their transactivating capacity. Therapeutic targets of JAK inhibitors and agents inhibiting the JAK/STAT signaling are indicated. This presentation was created in BioRender. Ni, X. (2025) https://BioRender.com/h10d557 (accessed on 29 January 2025).

The Janus kinase/signal transducer and activator of transcription (JAK/STAT) pathway is essential for interferon- or cytokine-signaling and T cell growth and function. It plays a critical role in the differentiation of helper T-cell subsets and immune system regulation. The JAK family—JAK1, JAK2, JAK3, and tyrosine protein kinase 2 (TYK2)—are intracellular non-receptor tyrosine kinases that initiate trans-phosphorylation of bound JAK proteins upon the binding of cytokines, interferons, or growth factors to their receptors. Activated JAK proteins create docking sites for STAT proteins (STAT1, STAT2, STAT3, STAT4, STAT5A, STAT5B, and STAT6), facilitating their dimerization and translocation to the nucleus, where they bind specific DNA sequences and lead to the transcription of cytokine-responsive genes (Figure 1). There are several negative regulators along the JAK/STAT pathway. Suppressors of cytokine signaling (SOCS) are the primary regulators of the JAK/STAT pathway, and act as pseudo-JAK substrates to inhibit JAK/STAT signaling. Protein inhibitors of activated STATs (PIASs) interact with activated STATs and inhibit their DNA binding and their transactivating capacity. Protein tyrosine phosphatases (PTPs) can dephosphorylate activated JAKs, STATs, or cytokine receptors.

Studies have found that the overactivation or dysregulation of the JAK/STAT pathway is linked to immunodeficiency, inflammatory diseases, autoimmunity, and tumorigenesis [4,5]. Consequently, therapeutic strategies targeting the JAK/STAT pathway have gained momentum, with increasing use of JAK/STAT inhibitors and agents with potent inhibitory effects on this pathway. Of interest, several FDA-approved JAK inhibitors for inflammatory diseases are now being explored for cancer treatment [3,4].

The constitutive activation of the JAK/STAT pathway may be a central driver of CTCL pathogenesis and progression [5]. To consolidate the current understanding of dysregulation of the JAK/STAT pathway and therapeutic implications in CTCL, we conducted a literature review of the PubMed, Web of Science, and ClinicalTrials.gov databases with the following keywords: cutaneous T-cell lymphoma, mycosis fungoides, Sézary syndrome, pathogenesis, JAK/STAT pathway, therapeutic strategies, JAK/STAT inhibitors, clinical response, and adverse effects. We highlighted six JAK inhibitors (golidocitinib, ruxolitinib, cerdulatinib, tofacitinib, upadacitinib, and abrocitinib) that have been evaluated in clinical and preclinical studies in CTCL. We also discuss five agents with inhibitory effects on the JAK/STAT pathway (ND16, ECPIRM [a 13-cis retinoic acid derivative], Tyrphostin AG490, Cucurbitacin, and Pimozide) that have been studied in vitro or in vivo in murine models of CTCL.

## 2. Dysregulation of the JAK/STAT Pathway in CTCL

The constitutive activation of the JAK/STAT pathway has been found in different subtypes of CTCL and implicated its pathogenesis. Overactivation of JAK/STAT signaling is often associated with aggressive clinical, inadequate response to therapy, and poor prognosis. Studies have suggested that the constitutive activation of the JAK/STAT pathway may be due to activating JAK/STAT mutations or inactivating mutations of negative regulators in the signaling cascade. Overactivation may also be caused by other mechanisms without activating mutations.

### 2.1. Activating Mutations of JAK/STAT Molecules

Activating mutations of JAK/STAT molecules have been found in CTCL. An early study reported that a JAK3A572V mutation related to constitutive JAK3 activation was found in one of thirty CTCL patients [6]. Other studies have confirmed that somatic gene mutations in JAK1 and JAK3 were implicated in dysregulated JAK/STAT signaling in CTCLs [7]. Point mutations and copy number amplifications were detected in JAK1 (0.9% of cases) and JAK3 (2.7%) in CTCL. JAK3 mutations were associated with advanced stage disease and large cell transformation [8]. The copy number gains of JAK2 were found in 13% of CTCL. In addition, point mutations and copy number amplifications were also detected in STAT3 (0.9%) and STAT5B (3.6%) in CTCL. The high incidences of copy number gains of STAT3 (60%) and STAT5B (60%) correlate with increased expression [7].

### 2.2. Inactivating Mutations of JAK/STAT Inhibitory Molecules

The overactivation of the JAK/STAT pathway can also be due to inactivating mutations of negative regulators along the JAK/STAT signaling cascade. As mentioned previously, there are several negative regulators along the JAK/STAT pathway, such as SOCSs, PIASs, and PIPs. The SOCS protein family plays a vital role in negatively regulating cytokine signaling. Notably, SOCS1 is the prototypical member, functioning in a classic negative feedback loop to inhibit cytokine-induced signaling [9]. Genomic analyses have identified SOCS1 as a recurrently deleted gene in MF, occurring even in the earliest stages of the disease [10]. In a genetically engineered CTCL mouse model, the allelic deletion of SOCS1 in the CD4 T cells of the skin led to an increase in CD3 and CD4 T cells in the skin and more activation of STAT3 compared with the control group [11].

### 2.3. JAK/STAT Overactivation Without Activating Mutation

Studies have found that JAK/STAT overactivation can also be caused by other mechanisms without activating mutations, such as epigenetic changes and gene transcript fusions. Han et al. found that increased miR-155-5p, miR-130b-3p, and miR-21-3p were correlated to decreased mRNA expression of SOCS, PIAS, and PTPN [12]. Transfecting MyLa and HuT78 cells with anti-miR-155-5p, anti-miR-21-3p, and anti-miR-130b led to a marked increase in SOCS protein levels and a significant reduction in activated STAT3 [12]. By RNAseq, our group and others have reported an oncogenic JAK3-INSL3 fusion transcript in Sézary syndrome [13,14]. Patients with a high-level expression of JAK3-INSL3 showed poorer 5-year survival than patients with a low-level expression. Further study found that knocking down JAK3-INSL3 expression in CTCL cells led to decreased expression of total JAK3, pJAK3, pSTAT1, pSTAT3, pSTAT5, pSTAT6, and NFκb proteins, which resulted in reduced cell proliferation and decreased colony formation in MJ and HH cell lines, as well as smaller tumor sizes in NSG xenograft mice [14]. Our results suggest that JAK3-INSL3 fusion may contribute to constituting the activation of the JAK/STAT pathway in CTCL and support the therapeutic targeting of the JAK/STAT pathway.

## 3. Therapeutic Strategies Targeting the JAK/STAT Pathway in CTCL

Targeting the JAK family kinase with small-molecule inhibitors has proven effective in the treatment of different types of diseases. To date, multiple JAK inhibitors have been approved for clinical use [15]. A few clinical trials and case studies have also shown promising results of JAK inhibitors in the treatment of CTCL patients. Preclinical studies have demonstrated the mechanisms underlying the efficacy of JAK/STAT inhibition in CTCL by JAK inhibitors or agents with inhibitory effects on the JAK/STAT pathway.

### 3.1. JAK/STAT Inhibitors

JAK inhibitors operate by impeding cytokine-mediated signaling via the JAK/STAT pathway. They bind to the active site of Janus kinases, inhibiting their enzymatic activity and subsequently preventing the phosphorylation of STAT proteins (Figure 1). These inhibitors can be grouped by their binding mode and interaction type with the amino acids in JAKs, into reversible (competitive) and irreversible (covalent) inhibitors. They are also categorized based on their targeting selectivity into pan-JAK inhibitors and selective inhibitors. To date, there are ten JAK inhibitors that have been approved by the FDA in the treatment of inflammatory diseases, including atopic dermatitis, rheumatoid arthritis, alopecia areata, vitiligo, ulcerative colitis, Crohn’s disease, ankylosing spondylitis, and graft-versus-host disease [15,16,17,18,19,20,21,22,23,24,25,26]. Four of these JAK inhibitors—ruxolitinib, fedratinib, pacritinib, and momelotinib—have been approved for myelofibrosis [17,20,24,26]. Ruxolitinib is also used for the treatment of polycythemia vera in adults [16].

There are no FDA-approved JAK inhibitors for CTCL; however, six JAK inhibitors have been clinically studied in CTCL (Table 1). In the following section, we highlight these six JAK inhibitors studied in both clinical and preclinical settings for CTCL.

#### 3.1.1. Golidocitinib

Golidocitinib is a potent and selective JAK1 inhibitor developed by Dizal Pharmaceutical Co., Ltd., for treating cancers [27,28]. Preclinical studies have demonstrated that it had strong anti-tumor activity in T-cell lymphoma cells in vitro and in tumor xenograft models in vivo.

The phase 1/2 study with golidocitinib in r/r PTCL (JACKPOT8, NCT04105010) demonstrated a favorable benefit–risk profile with an objective response rate (ORR) of 44.3% (39 of 88 patients), including 21 (24%) patients having a complete response and 18 (20%) having a partial response [28]. The safety analysis revealed that the most common grade 3–4 treatment-emergent adverse events associated with the drug were reductions in neutrophil count (29%), white blood cell count (26%), lymphocyte count (21%), and platelet count (20%). These adverse effects were clinically manageable and reversible. Serious treatment-related adverse events occurred in approximately 24% of patients. In June 2024, golidocitinib received conditional approval in China for the treatment of adult patients with relapsed or refractory peripheral T-cell lymphoma (r/r PTCL) who had undergone at least one prior line of systemic therapy [27].

A phase 2 trial of golidocitinib monotherapy for r/r PTCL (JACKPOT27; NCT06511895) is currently enrolling patients. Another phase 2 trial exploring golidocitinib as maintenance therapy after standard first-line treatment for PTCL (JACKPOT26; NCT06511869) is ongoing. Additionally, an investigator-initiated phase 2 study combining golidocitinib with CHOP as a front-line therapy for PTCL (NCT05963347) has recently begun.

#### 3.1.2. Ruxolitinib

Ruxolitinib is a first-generation JAK inhibitor that primarily inhibits JAK1, JAK2, and JAKV617F, and blocks cytokine signaling through the JAK/STAT pathway. Use of oral ruxolitinib was approved in 2011 [17] for the treatment of high-risk myelofibrosis [17] and for the treatment of polycythemia vera in 2014 [16]. Oral ruxolitinib has also been approved for steroid-refractory acute and chronic graft-versus-host disease (GVHD) [29,30]. Topical ruxolitinib 1.5% cream was approved for atopic dermatitis (AD) [31] and for vitiligo [32].

A phase 2 study investigated oral ruxolitinib in patients with r/r PTCL and MF (NCT02974647) [33]. In the study, patients were stratified into the following three biomarker-defined cohorts: Cohort 1—patients with activating JAK and/or STAT mutations; Cohort 2—patients with more than 30% pSTAT3 expression among tumor cells by immunohistochemistry; and Cohort 3—patients lacking adequate tissue for evaluation or with no tissue for assessment. Patients received ruxolitinib at a dose of 20 mg orally twice daily until disease progression. In the trial, one out of seven MF patients whose tumors showed pSTAT3 expression in 20% of cells demonstrated a PR to ruxolitinib lasting greater than 18 months. For r/r PTCL patients, clinical benefit rates (CBR) in Cohorts 1, 2, and 3 were 53%, 45%, and 13%, respectively. Exploratory analysis indicated that the expression levels of phosphorylated S6 in less than 25% of tumor cells correlated with a response to ruxolitinib. These findings suggest the potential efficacy of oral ruxolitinib in patients with activating JAK/STAT mutations and/or high expression of pSTAT3.

Common adverse events of ruxolitinib in this study included anemia and thrombocytopenia, which generally improved over time. The medium-term immune suppression and potential long-term decreases in natural killer cell function occurred in patients with prolonged use of ruxolitinib. The most common nonhematologic adverse events included ecchymosis, headache, dizziness, abdominal pain, fatigue, and diarrhea.

The exploratory studies are ongoing to test the combinational effects of ruxolitinib with other agents. An in vitro study showed that ruxolitinib in combination with a histone deacetylase (HDAC) inhibitor, such as reminostat, showed synergistic antitumor effects in CTCL cell lines, MyLa and SeAx [34].

#### 3.1.3. Cerdulatinib

Cerdulatinib is a small-molecule reversible ATP competitive inhibitor of JAK family members and SYK [35]. Cerdulatinib can also block the cell cycle by inhibiting RB phosphorylation and down-regulating cyclin E. As a dual inhibitor of JAK and SYK pathways, cerdulatinib has shown efficacy in patients with r/r PTCL or CTCL [36].

Results from a phase 2a study evaluated cerdulatinib in 98 patients with r/r PTCL (*n* = 61) or r/r CTCL (*n* = 37) after ≥1 prior systemic therapy [36]. Patients received cerdulatinib at 30 mg orally twice daily. The ORR was 35% in both PTCL and CTCL cohorts. In CTCL, MF had greater activity (ORR 45%, CR 9%) than SS (ORR 17%). Beyond its impact on tumor response, a significant proportion of CTCL patients experienced rapid relief from pruritus, a distressing symptom commonly associated with these conditions [36]. The dual mechanism of action of cerdulatinib may offer a broader therapeutic profile by both controlling tumor progression and providing symptomatic relief, warranting further exploration in larger trials [36].

Common grade 3+ adverse events included lipase (21%) and amylase (18%) increases, diarrhea, and neutropenia. These findings support cerdulatinib tolerability and clinical activity in T-cell lymphomas. These results also suggest that with a dual mechanism of action targeting both JAK and SYK pathways, cerdulatinib has the potential to address both the underlying lymphoma and also symptomatic relief, highlighting its broad therapeutic potential in these challenging hematologic malignancies [36].

#### 3.1.4. Tofacitinib

Tofacitinib is a pan-JAK inhibitor and works by inhibiting the JAK1, JAK2, and JAK3 enzymes and cytokine-mediated signals through the JAK-STAT pathway. It has been FDA-approved for use in rheumatoid arthritis, psoriatic arthritis, ulcerative colitis in adults, polyarticular-course juvenile idiopathic arthritis in people 2 years of age and older, and ankylosing spondylitis in adults [18,37]. Tofacitinib is available in 5 mg and 10 mg tablets and as an 11 mg extended-release tablet for oral use in adults. It is also available as a 1 mg/mL oral solution for children aged 2 years and older [15]. Reported adverse effects of tofacitinib include upper respiratory tract infection, nasopharyngitis, diarrhea, gastroenteritis, nausea, headache, elevated cholesterol levels, rash, hypertension, anemia, and herpes zoster [15].

Multiple preclinical studies have been conducted to explore the effects of tofacitinib in CTCL and other T-cell lymphomas. Ando et al. reported that activation of the JAK3/STAT5 was observed in EBV-positive T- and NK lymphoma cell lines as well as patient samples [38]. They found that tofacitinib effectively suppressed proliferation, induced G1 cell-cycle arrest, and reduced EBV LMP1 and EBNA1 expression in these cells. Tofacitinib also inhibited tumor growth in a murine xenograft model [38]. These results support tofacitinib as a potential therapeutic agent for EBV-associated T- and NK-cell lymphomas.

**Table 1:** Clinical studies of JAK inhibitors in CTCL.

Drug(s)	Target(s)	Disease(s)	Study Phase	Interventions	Status	Results	Clinical Trials	Ref.
Golidocitinib	JAK1	r/r PTCL (*n* = 88): received ≥ systemic treatment,	1/2	150 mg QD orally, on 28-day cycle	Completed, Conditional approval by NMPA	ORR: 44.3%, with a 24% CR and 20% PR	NCT04105010	[27,28]
		r/r PTCL: Cohort 1: Newly enrolling. Cohort 2: Completed study still benefiting	2	150 mg QD orally, on 28-day cycle	Recruiting	n/a	NCT06511895	[27]
		PTCL	2	150 mg QD orally, on 28-day cycle	Ongoing	n/a	NCT06511869	[27]
Golidocitinib + CHOP	JAK1 +	PTCL	2	150 mg QD orally, on 28-day cycle, plus CHOP	Recruiting	n/a	NCT05963347	[27]
Ruxolitinib	JAK1, JAK2, JAK2V617F	MF (*n* = 7), PTCL (*n* = 45): Cohort 1: with JAK/STAT mutations; Cohort 2: >30% pSTAT3; Cohort 3: others	2	20 mg BID orally on 28-day cycles	Part of results reported, still recruiting	CBR in MF: 14% (1/7), CBR in PTCL: Cohort 1–53% (10/19); Cohort 2–45% (5/11); Cohort 3–13% (2/15)	NCT02974647	[33]
Cerdulatinib	JAK1, JAK2, JAK3, TYK, SYK	r/r PTCL (*n* = 61), r/r CTCL (*n* = 37), ≥1 prior systemic therapy	1/2a	30 mg BID orally	Completed	r/r PTCL: AITL/TFH–ORR 55%, CR 41%; r/r CTCL: MF: ORR 45%, CR 9%, SS: ORR 17%, CR 0%	NCT01994382	[36]
Tofacitinib	JAK3, JAK2, JAK1	Early-stage CTCL, IA, IB, and IIB	2	2% cream, topically	Initiated	n/a	NCT06698822	n/a
Tofacitinib + Chidamide	JAK3, JAK2, JAK1, +HDACs	r/r ENKTCL	2	Tofacitinib–orally 10 mg daily. Chidamide–orally 20 mg twice weekly	unknown	n/a	NCT03598959	n/a
Upadacitinib	JAK1, JAK2, JAK3	Erythrodermic MF	Case study	Orally 15 mg daily, 16 weeks	completed	CR	None	[39]
		MF	Case study	Orally, treatment for 2 weeks	completed	Improved post-inflammatory hyperpigmentation and pruritus	None	[40]
Acrocitinib	JAK1	Recalcitrant folliculotropic MF (T1bNxM0B0, mSWAT: 3)	Case study	100 mg QD Orally, 3 months	completed	Improved mSWAT (>50%) and the skin lesions, negative lymphadenopathy after 6 months	None	[41]

r/r PTCL: relapsed or refractory peripheral T-cell lymphomas; r/r CTCL: relapsed/refractory cutaneous T-cell lymphomas; MF: mycosis fungoides; SS: Sézary syndrome; r/r ENKTCL: relapsed/refractory extranodal NK/T-cell lymphomas; QD: once a day; BID: twice a day; CHOP: cyclophosphamide, doxorubicin hydrochloride (hydroxydaunorubicin), vincristine sulfate (Oncovin), and prednisone; NMPA: National Medical Products Administration; HDACs: histone deacetylases; CBR: Clinical Benefit Rate; ORR: objective or overall response rate; CR: complete response; PR: partial response.

It is known that malignant T cells can lead to skin barrier defects in CTCL by orchestrating changes in the microenvironment. A study investigating these pathological processes found that areas adjacent to TOX-positive T cells in CTCL skin lesions exhibited increased trans-epidermal water loss (TEWL) and compromised the expression of skin barrier proteins like filaggrin and filaggrin-2 [42]. Consequently, treatment with tofacitinib increased filaggrin expression in lesional skin from patients with MF. This suggests that JAK inhibitors could provide novel treatment options for patients with advanced CTCL and a compromised skin barrier [42].

Tofacitinib is being evaluated clinically for the treatment of CTCL. A phase 1/2 study of tofacitinib combined with chidamide (a HDAC inhibitor) in treatment of EN/NK/T-cell lymphoma (NCT03598959) and a phase 2 study of 2% tofacitinib cream in early-stage CTCL patients (IA, IB, and IIA) has been initiated (NCT06698822).

#### 3.1.5. Upadacitinib

Upadacitinib is a second-generation, selective JAK inhibitor that targets JAK 1, 2, and 3 enzymes, with preferential inhibition of JAK1. It is FDA approved for the treatment of autoimmune and inflammatory conditions, including moderate to severe rheumatoid arthritis, psoriatic arthritis, AD, moderate to severe Crohn’s disease, ankylosing spondylitis, non-radiographic axial spondylarthritis, and moderate to severe ulcerative colitis [21]. Upadacitinib is available as 15 mg and 30 mg extended-release tablets for oral administration.

While large-scale trials with upadacitinib specific to CTCL are currently lacking, two case reports illustrate a good response or symptoms improving in MF patients. One patient with erythrodermic MF [39] received oral upadacitinib 15 mg daily for 16 weeks and demonstrated a complete response with dramatic improvement in generalized pruritus, erythema, and scale (body surface area involvement decreased to <10%). Another patient with MF reported significant improvement in post-inflammatory hyperpigmentation and pruritus following upadacitinib treatment for 2 weeks [40]. These findings suggest a promising role for upadacitinib in managing CTCL. Future clinical studies are needed to establish the safety and efficacy in CTCL patients.

#### 3.1.6. Abrocitinib

Abrocitinib is an oral small-molecule inhibitor of Janus kinase 1 (JAK1) being developed by Pfizer. It preferentially inhibits the cytokine-induced phosphorylation of STAT by JAK pairs that include JAK1 [23]. It was FDA approved in 2022 (NCT03627767, NCT03720470) for the treatment of moderate-to-severe AD [15,23]. The recommended dosage is 100 or 200 mg once daily. Abrocitinib was associated with a dose-dependent reduction from baseline in serum levels of inflammatory markers (IP-10 and CRP). Reported adverse effects of abrocitinib include reduced platelet counts and elevated lipoprotein and cholesterol. There are few studies of abrocitinib treatment for other diseases including CTCL.

Liu et al. reported a case of recalcitrant folliculotropic MF successfully treated by abrocitinib [41]. The patient, with refractory folliculotropic MF, exhibited a rapid and significant response to the selective JAK1 inhibitor abrocitinib as an adjunct therapy alongside interferon and phototherapy with mild adverse events. These findings pose the potential of selective JAK1 inhibitors as a complementary therapy to achieve a rapid response in refractory folliculotropic MF, which could be further validated in future clinical trials.

### 3.2. Agents with Potent Inhibitory Effects on the JAK/STAT Pathway in CTCL

Besides small molecule JAK inhibitors, many preclinical studies have assessed other agents with potent inhibiting effects on the JAK/STAT pathway in CTCL. We discuss five of them listed below.

#### 3.2.1. ND-16

ND-16, a novel nilotinib derivative, has demonstrated anticancer effects on CTCL cells [43]. It selectively inhibits JAK2 in CTCL H9 cells. Further investigation revealed that ND-16 inhibited downstream cascades of JAK2, including STATs, PI3K/AKT/mTOR, and MAPK pathways. It also regulated Bcl-2 family members and CDKs/cyclins, leading to cell apoptosis and cell cycle arrest at S-phase. ND-16 holds promise as a potential therapy for the management of CTCL.

#### 3.2.2. ECPIRM

Retinoids are important agents for CTCL treatment, but their side effects and drug resistance have limited their clinical application. Yang et al. found that ECPIRM, a new 13-cis retinoic acid derivative, inhibited cell proliferation, induced apoptosis, and led to cell cycle arrest in HuT 78 cells by down-regulation of JAK1 activation and subsequent STAT3/5 signaling [44]. ECPIRM also induced G0/G1 phase arrest by decreasing the expression of cyclinD1, cyclin E, CDK2, and CDK4 while increasing p21.

#### 3.2.3. Tyrphostin AG-490

Tyrphostin AG-490 is a ATP-competitive JAK inhibitor [45]. The study conducted by Kirken et al. found that Tyrphostin AG-490 inhibited cytokine-mediated JAK3/STAT5A/B signal transduction and cellular proliferation of antigen-activated human T cells [46]. Nielsen et al. tested effects of Tyrphostin AG-490 in MF-derived cell line and found that it blocked the constitutive activation of slow migrating isoform of STAT3 and inhibited spontaneous as well as IL-2-induced growth of MF cells [47]. Eriksen et al. investigated the effects of Tyrphostin AG-490 in leukemic Sézary cells and found it inhibited STAT3 activation and the growth of leukemic Sézary cells [48]. Tyrphostin AG-490 also down-regulated the expression of the IL-2 receptor and induced apoptosis in Sézary cells.

#### 3.2.4. Cucurbitacins

Cucurbitacins are a family of plant-derived triterpenoids and have been reported to inhibit cancer cell proliferation via interference with the JAK/STAT pathway. Studies have shown promising results with cucurbitacin I and E in CTCL cells in vitro [49,50]. Incubation of SeAx cells with cucurbitacin I resulted in a time- and dose-dependent decrease in pSTAT3, while incubation of leukemic Sézary cells with cucurbitacin I induced cell apoptosis [49]. Cucurbitacin I and E decreased the cell viability of SeAx and HuT 78 cells, as well as primary Sézary cells [50].

#### 3.2.5. Pimozide

Pimozide, a neuroleptic drug, has been studied in cancers due to its mechanism of action inhibiting the JAK/STAT signaling pathway, specifically by suppressing the phosphorylation/activation of STAT proteins, particularly STAT5 [51]. A study reported that pimozide had inhibitory effects on STAT5B in primary PTCL patient-derived cell lines, and induced cell apoptosis via TRAIL/DR4-dependent extrinsic apoptotic pathway [52].

### 3.3. Combinational Strategies of JAK/STAT-Targeted Therapies

Although the JAK/STAT pathway is a good therapeutic target, the effectiveness of the JAK/STAT inhibition may be limited by the patient-specific JAK/STAT status. Moreover, the pathogenesis of CTCL is complex, involving genomic aberration, epigenetic changes, immune suppression, as well as persistence of immortal lymphoma cells. As a result, JAK/STAT-targeted therapy alone may be insufficient to treat or cure the disease. Combining JAK/STAT-targeted therapy with other targeted therapies or immunotherapy may achieve better clinical responses or even a cure. Numerous preclinical/clinical studies have demonstrated the synergistic effects of JAK inhibitors when combined with other therapies in CTCL.

#### 3.3.1. Combining JAK Inhibitors with Epigenetic Modifiers

In contrast to gene mutations, epigenetic changes are reversible and serve as an attractive target for cancer therapy. Multiple epigenetic modifiers such as histone deacetylase (HDAC) inhibitors have shown anticancer activity in CTCL. Two HDAC inhibitors, suberoylanilide hydroxamic acid (SAHA) vorinostat and romidepsin, have been FDA approved for the treatment of CTCL. Another HDAC inhibitor, resminostat, is currently in a phase 2 study for SS (NCT02953301). A study has investigated the efficacy of ruxolitinib in combination with resminostat in CTCL cell lines [34]. Ruxolitinib or resminostat alone showed some degrees of inhibition of cell viability in MyLa and SeAx cells, but the combination of the two compounds further inhibited cell proliferation and induced apoptosis in both cell lines. Of importance, the combination inhibited phosphorylation of STAT3, Akt, ERK1/2, and JNK in these cells. These results suggest that the combination of a JAK inhibitor and HDAC inhibitor had a strong synergistic anti-tumor effect in CTCL cell lines and may represent a promising novel therapeutic modality for CTCL patients.

As mentioned above, a phase 1/2 study is ongoing to investigate the efficacy and safety of tofacitinib combined with chidamide, another HDAC inhibitor, in patients with relapsed and refractory extranodal NK/T cell lymphoma (NCT03598959).

#### 3.3.2. Combining JAK Inhibitors with Immunotherapy

Immune suppression is accompanied with the disease progression, treatment resistance, and poor prognosis in CTCL [53]. Programmed death 1 (PD1)/PD ligand 1 (PD-L1) promotes cancer cell evasion from immune surveillance, thus it has been studied as an immunotherapy target. Studies have shown that the anti-PD-L1 antibody, durvalumab, was able to re-program M2 macrophages and boost antitumor functions against CTCL cells [54]. Querfeld et al. also reported that durvalumab had good clinical activity in refractory and advanced CTCL patients in a phase 1/2 trial [55]. However, many patients do not respond or are resistant to this therapy. The JAK/STAT pathway is highly correlated with PD1/PD-L1 expression [56], thus is likely associated with resistance to anti-PD1/PD-L1 therapy. Clinical trials are ongoing investigating the JAK/STAT inhibitors in combination with anti-PD-1/PD-L1 therapies in solid tumors and hematological malignancies [56]. Zak et al. reported a phase 1 clinical trial of ruxolitinib combined with an anti-PD1 antibody, nivolumab, in Hodgkin lymphoma patients. They found the combination showing the best overall response rate [57]. The STAT3 activation was correlated with increases in expression of PD-L1 in NLTCL and PTCL [58,59]; thus, the combination of JAK/STAT inhibitors with PD1/PD-L1 antibodies could be a promising therapeutic strategy to explore.

#### 3.3.3. Combining JAK Inhibitors with Modulators of Apoptosis

Defective apoptosis drives the immortal growth of lymphoma cells and is closely associated with elevated levels of Bcl-2 and Bcl-XL. Multiple effective therapies work by directly or indirectly inducing cell apoptosis. The strategy of targeting and reducing Bcl-2/Bcl-xL has proven effective in promoting cell apoptosis. Zhang et al. investigated the combination of ruxolitinib with navitoclax, a Bcl-2/Bcl-xL inhibitor in PBMCs from ATLL patients and ATLL murine models [60]. They found that ruxolitinib and navitoclax demonstrated modest antitumor activity individually, but their combination significantly suppressed the ex vivo proliferation of patients’ PBMCs, markedly reduced tumor burden, and extended survival in a murine model. These findings highlight the potential therapeutic advantage of a combination approach targeting both JAK/STAT signaling and the antiapoptotic proteins Bcl-2/Bcl-xL in patients with ATLL [60].

#### 3.3.4. Combining Different JAK Inhibitors

As discussed above, different JAK inhibitors target different JAK member(s), and their anti-tumor effects vary by disease and by patient-specific JAK/STAT activation status. Gomez-Arteaga et al. reported a successful case of combining tofacitinib (a pan-JAK inhibitor) with ruxolitinib (a JAK1/2 inhibitor) to treat refractory T-cell prolymphocytic leukemia (T-PLL) harboring a JAK3 mutation [61]. Their study suggested that a combination of JAK inhibitors may be a more effective therapeutic strategy.

#### 3.3.5. Combinational Strategy Against Multiple Targets

Since the pathogenesis of CTCL is complex, a combinational therapeutic strategy against multiple targets should be a great strategy for CTCL. Yumeen et al. highlighted the potential roles of combination approaches using JAK, Bcl-2, bromodomain and extra-terminal domain (BET), and HDAC inhibition in advanced CTCL [62]. They investigated the effects of JAK inhibition alone or in combination with other targeting agents on peripheral blood malignant CTCL isolates and established CTCL cell lines. Regardless of single-agent sensitivity, JAK inhibition significantly enhanced the cytotoxic effects on malignant cells when combined with inhibitors targeting Bcl-2, BET, HDAC, or the proteasome. Among these combinations, JAK and Bcl-2 inhibition showed the most potent cytotoxic effect against CTCL cells, acting through both intrinsic and extrinsic apoptosis pathways. Interestingly, JAK inhibition reduced Bcl-2 expression in high-responder samples, suggesting a potential mechanism for this synergistic effect. These findings underscore the impactful role of JAK inhibition in CTCL and suggest that combining JAK inhibitors with other targeted therapies could yield broader and more effective cytotoxic responses in CTCL patients. JAK inhibition synergistically potentiates Bcl-2, BET, HDAC, and proteasome inhibition in advanced CTCL. These preclinical results suggest that the combination targeted drug approaches may be a means of clinical utilization in the treatment of CTCL [62].

## 4. Concerns About the Potential Risk of Relapsing or Emerging CTCL Associated with JAK Inhibitor Use

Recently, several cases have reported CTCL relapsing or emerging following JAK inhibitor treatment [63,64,65,66,67]; thus, the risk of developing/worsening malignancies including CTCL with the use of JAK inhibitors draws attention. Here, we present a summary of these reports in Table 2 and discuss them in detail below.

**Table 2:** CTCL cases following JAK inhibitor treatment.

Drug (s)	Clinical Info and Original Disease	Previous Treatment	Administration	Results	Ref.
Updacitinib	Female, 74 yrs, rheumatoid arthritis (RA) for 4 years	Sulfasalazine, methotrexate, iguratimod, prednisolone, tocilizumab for about 3 years, followed by peficitinib for 1 year	Upadacitinib, 7.5 mg/d, 2 weeks	Lymphomatoid papulosis (LyP) at extremities	[67]
Tofacitinib	Male, 42 yrs, erythema elevatum diutinum for 10 years	Dapsone, adalimumab, mycophenolate mofetil, and sulfasalazine	Tofacitinib, 8 weeks	LyP in skin and eye	[66]
Baricitinib	Male, 78 yrs, seronegative RA for 2 years	methotrexate, tocilizumab, bucillamine, adalimumab and betamethasone for 2 years	Baricitinib added to betamethasone, 7 months	Sézary syndrome (SS)	[64]
Ruxolitinib	Case 1: male, 63 yrs, MF with large-cell transformation for 13 years (T3N0M0B0) Case 2: Female, 53 yrs, folliculotropic MF with large-cell transformation for 13 years (T2N0M0B0)	Multiple treatments followed by allogeneic stem cell transplantation (allo-HSCT)	Case 1 (GVHD): IgIV, prednisone, ruxolitinib (20 mg/d 12 months) Case 2 (GVHD) IgIV, prednisone, ruxolitinib (30 mg/d 9 months)	Case 1: Relapse of transformed CTCL (T4N0M0B0) Case 2: SS (T4NxM0B2)	[65]
Baricitinib + Upadacitinib	Female, 26 yrs, early MF misdiagnosed as AD for 7 years, with a history of asthma and AD in childhood	Cyclosporine followed by dupilumab for 2 months	Baricitinib, 4 mg/d, 6 weeks; followed by upadacitinib, 15 mg/d, then by 30 mg/d, several weeks	IVA2 MF	[63]

RA: rheumatoid arthritis; LyP: lymphomatoid papulosis; MF: mycosis fungoides; SS: Sézary syndrome; GVHD: graft-versus-host disease.

### 4.1. Upadacitinib- or Tofacitinib-Associated Lymphomatoid Papulosis

In 2022, Linuma et al. reported the first case—a 74-year-old woman developed lymphomatoid papulosis (LyP) two weeks after starting upadacitinib for rheumatoid arthritis (RA) [67]. Drug-induced LyP has been previously reported, including cases induced by cyclosporine and TNF-inhibitors (infliximab and adalimumab) [68,69,70], but this is the first instance of LyP linked to upadacitinib. Although the precise mechanism is unclear, the authors speculated that immunosuppression due to JAK1-selective inhibition by upadacitinib may underlie the susceptibility to LyP in the patient. However, owing to her treatment history for RA, it was difficult to incriminate upadacitinib as the sole cause of LyP. Upadacitinib is widely used for inflammatory diseases, such as psoriatic arthritis and AD; therefore, the authors reminded dermatologists to be aware that LyP could be a potential adverse event. Further studies are needed to gain a deeper understanding of the pathological mechanisms and clinicopathological features of upadacitinib-associated LyP.

In the same year, Knapp et al. reported a patient with refractory erythema elevatum diutinum who developed cutaneous LyP with ocular involvement following 8 weeks of tofacitinib treatment [66]. In fact, previous studies have implicated the activation of the JAK-STAT pathway in the development of LyP. Maurus et al. conducted a study of 12 patients with CD30^+^ lymphoproliferative disorders, including six patients with LyP [71]. They found an increased frequency of JAK/STAT activating mutations, and four of the six patients with LyP had single-nucleotide variants in various STAT proteins, with three patients having mutations in STAT3 or STAT6 [71]. The occurrence of LyP in a patient receiving tofacitinib prompts further investigation into the relationship between JAK/STAT inhibition, lymphoproliferative disorders, and immune-mediated adverse events.

### 4.2. Baricitinib-Associated Sézary Syndrome

Baricitinib is a JAK inhibitor selective for JAK1 and JAK2 and used for the treatment of RA, alopecia areata, and AD [19]. Saito et al. in 2023 reported a case of SS in a patient during treatment with baricitinib for seronegative RA [64]. Seven months into baricitinib treatment, the patient presented with erythroderma, elevated lymphocyte counts, and Sézary cells, diagnosed with SS. The patient achieved partial remission after discontinuing baricitinib and initiating therapies such as mogamulizumab, bexarotene, and phototherapy.

### 4.3. Relapses of CTCL After Ruxolitinib Treatment of Chronic Graft-Versus-Host Disease

Cohen et al. recently reported two cases of severe relapses of CTCL after treatment of chronic graft-versus-host disease (cGVHD) with ruxolitinib [65]. Two patients with advanced MF relapsed aggressively after using ruxolitinib for cGVHD management following allogeneic hematopoietic stem cell transplantation (allo-HCST) [65]. These relapses were marked by erythroderma and extensive skin tumors, with blood involvement in one case.

### 4.4. Worsening of MF Following Treatment of Baricitinib and Upadacitinib

Lamolet et al. reported that a patient with CTCL had their disease possibly worsened by treatment with JAK inhibitors [63]. The patient had a history of AD in childhood and was misdiagnosed with early MF as a recurrence of AD. The patient was treated with baricitinib and upadacitinib, experiencing brief improvement before developing worsening lesions, changing symptoms, and inguinal lymphadenopathy. This case highlights the longstanding diagnostic challenge of early MF and underscores the importance of a careful differential diagnosis between AD and early MF.

As discussed earlier, JAK inhibitors have been considered potential candidates for the treatment of CTCL. However, the cases reporting CTCL relapse or onset following JAK inhibitor treatment highlight the need for further investigation to evaluate this risk in the context of CTCL treatment. Careful monitoring and follow-up are recommended, particularly for patients with RA undergoing treatment with JAK inhibitors (i.e., upadacitinib and baricitinib). Furthermore, the cautious use of ruxolitinib to manage GVHD is warranted in patients who have received allo-HCST for advanced-stage CTCL. While it remains unclear whether JAK inhibitors directly induce the development, relapse, or exacerbation of CTCL, these possibilities warrant serious investigation.

## 5. Summary

Despite the diverse treatment landscape for CTCL, from skin-directed treatment to systemic therapies, patients with advanced or refractory disease still have limited options and often poor outcomes. The findings presented in this review underscore that the therapeutic targeting of the JAK/STAT pathway is a promising strategy in the management of CTCL.

JAK inhibitors like golidocitinib, ruxolitinib, and cerdulatinib have shown potent anti-tumor effects in early-phase clinical studies. Golidocitinib, a selective JAK1 inhibitor, demonstrated substantial anti-tumor activity in r/r PTCL [28], which suggests potential utility in CTCL given the overlapping molecular features between these lymphomas. Ruxolitinib, a JAK1/2 inhibitor, showed good clinical responses in both MF patients and PTCL patients [33]. Cerdulatinib, with dual inhibition of JAK and SYK pathways, not only exhibited efficacy in controlling r/r CTCL and r/r PTCL but also provided rapid relief from pruritus, addressing one of the most distressing symptoms for CTCL patients [36]. Upadacitinib, a selective JAK1 inhibitor, also demonstrated clinical potential in MF, with two case reports noting improvements in symptoms like pruritus [39,40]. Larger-scale studies are necessary to establish its efficacy and long-term safety profile in CTCL. Tofacitinib, a pan-JAK inhibitor, showed promise in enhancing skin barrier integrity in CTCL patients by up-regulating barrier proteins like filaggrin, which may provide dual benefits by targeting both the malignant T cells and alleviating skin-related symptoms in CTCL [42].

Novel agents targeting the JAK/STAT and related pathways, such as ND-16, ECPIRM, tyrphostin AG490, cucurbitacin, and pimozide, have shown efficacy in preclinical studies and represent exciting future directions for CTCL therapy. These agents offer additional mechanisms, such as cell cycle arrest and down-regulation of STAT activation, that may complement JAK inhibition and further enhance therapeutic outcomes. Integrating these emerging therapies into the treatment paradigm may ultimately expand options for patients with CTCL, particularly those with refractory or relapsed disease.

While JAK inhibitors show promising results, they are not without risks and concerns. Common adverse events include immunosuppression, hematologic toxicities, infections, and increased risk of malignancies. Therefore, risk–benefit assessments are critical, particularly in elderly or immunocompromised patients who may be more susceptible to these adverse effects. Moreover, it remains essential to define biomarkers that predict treatment response, allowing for personalized therapy and minimizing unnecessary exposure to potential toxicities.

## 6. Conclusions

The use of JAK inhibitors in CTCL therapy shows great promise but remains in the early stages of clinical validation. The preliminary data suggest their efficacy, particularly in molecularly defined subtypes, but larger clinical trials are necessary to confirm their effectiveness and safety in CTCL. Moreover, combination strategies incorporating JAK inhibitors with other targeted therapies could offer a more comprehensive approach, addressing both the malignancy and its associated symptoms. Moving forward, optimizing patient selection through molecular profiling, evaluating long-term safety, and exploring combination regimens will be essential in establishing JAK inhibitors as a good option in CTCL treatment.
